# The Hippo pathway acts via p53 and microRNAs to control proliferation and proapoptotic gene expression during tissue growth

**DOI:** 10.1242/bio.20134317

**Published:** 2013-06-20

**Authors:** Wei Zhang, Stephen M. Cohen

**Affiliations:** 1 Institute of Molecular Cell Biology, 61 Biopolis Drive, Singapore 138673; 2 Department of Biological Sciences, National University of Singapore, 14 Science Drive 4, Singapore 117543

**Keywords:** p53, Hippo pathway, Yorkie/YAP, *reaper*, MicroRNA, Apoptosis, Proliferation

## Abstract

The Hippo pathway has a central role in coordinating tissue growth and apoptosis. Mutations that compromise Hippo pathway activity cause tissue overgrowth and have been causally linked to cancer. In *Drosophila*, the transcriptional coactivator Yorkie mediates Hippo pathway activity to control the expression of cyclin E and Myc to promote cell proliferation, as well as the expression of *bantam* miRNA and DIAP1 to inhibit cell death. Here we present evidence that the Hippo pathway acts via Yorkie and p53 to control the expression of the proapoptotic gene *reaper*. Yorkie further mediates *reaper* levels post-transcriptionally through regulation of members of the miR-2 microRNA family to prevent apoptosis. These findings provide evidence that the Hippo pathway acts via several distinct routes to limit proliferation-induced apoptosis.

## Introduction

Cell proliferation is intimately linked with cell death. Cues that drive cell growth and division also induce apoptosis (Pelengaris et al., 2002). In an abnormal cell proliferation scenario, such as cancer, cells adopt a variety of strategies to overcome cell death (Hanahan and Weinberg, 2011). Many signaling pathways that drive tissue growth have been found to coordinate cell proliferation and apoptosis during animal development. Defects in these pathways quite often cause tissue overgrowth or cancer.

The Hippo pathway is one such signaling pathway, acting as a negative growth regulator. Mutations in several members of the pathway lead to tumorigenesis, implicating them as tumor suppressors (Cai et al., 2010; Pan, 2010). The core pathway comprises a kinase cascade including the Hippo and Warts kinases with their adaptors Salvador (Sav) and Mats (Moberg et al., 2001; Tapon et al., 2002; Harvey et al., 2003; Udan et al., 2003; Wu et al., 2003). Several proteins have been implicated as upstream regulators of this kinase cascade by genetic studies, including Merlin/NF2, Expanded and the atypical cadherin, Fat (Hamaratoglu et al., 2006; Silva et al., 2006; Yu et al., 2010). Downstream, the Warts kinase directly phosphorylates and inactivates transcriptional coactivators including YAP, TAZ, and in *Drosophila*, Yorkie (Yki) (Huang et al., 2005; Zhao et al., 2007; Zhao et al., 2008; Zhao et al., 2010). YAP/TAZ and Yki function to promote cell proliferation and inhibit apoptosis. These proteins possess no DNA binding activity and therefore bind to transcription factors including Scalloped/TEAD to activate their targets.

Genetic studies have identified functional targets of Yki with positive roles in cell proliferation, including cycE and Myc, as well as negative regulators of apoptosis, including the *Drosophila* Inhibitor of Apoptosis Protein (DIAP1) and the antiapoptotic microRNA *bantam* (Moberg et al., 2001; Moberg et al., 2004; Huang et al., 2005; Nolo et al., 2006; Thompson and Cohen, 2006; Neto-Silva et al., 2010). These studies have suggested that the Hippo pathway can balance proliferative drive with limitation of proliferation-induced apoptosis. This combination of roles may also explain the potency of mammalian YAP in control of tissue growth and its ability to induce cancer when overexpressed (Dong et al., 2007; Pan, 2010).

The tumor suppressor p53 is another key regulator coordinating cell division and cell death. Activation of p53 by the DNA damage checkpoint or other cell cycle abnormalities, leads to growth arrest, and initiates apoptosis. Activated p53 binds DNA and directs expression of downstream genes including p21, which inhibits the activity of cyclin–CDK complexes and activates cell cycle checkpoints to halt cell division (Gartel and Radhakrishnan, 2005). In addition, p53 promotes transcription of the proapoptotic genes Bax, PUMA and Apaf-1 to induce cell death. Recent studies in human cells have identified the ASPP1 protein (apoptosis-stimulating protein of p53-1) as a key mediator of p53-induced apoptosis (Aylon et al., 2010; Vigneron et al., 2010). The Hippo pathway kinase Lats, the mammalian homolog of Warts, phosphorylates ASPP1 and forms a complex with ASPP1 and p53 to activate the proapoptotic transcription program. Phosphorylation of ASPP1, however, can be antagonized by another Lats substrate YAP.

The upstream control of the apoptosis program is conserved in *Drosophila*, with p53 serving as a mediator of the DNA damage checkpoint. However, the effector program involves a set of insect-specific proapoptotic genes: *reaper, head involution defective (hid), grim and sickle (skl)* (Steller, 2008). The proapoptotic activity of these four proteins results from their ability to bind and inactivate DIAP, which in turn inhibits caspases. In mammals the corresponding functions are provided by Apaf-1 to cleave and activate caspases instead of derepression of caspases (Pop et al., 2006). Previous studies in *Drosophila* have shown that the *bantam* microRNA acts to repress *hid* to limit proliferation induced apoptosis (Brennecke et al., 2003). *bantam* mediates interaction between the EGFR and Hippo growth control pathways (Herranz et al., 2012). microRNAs of the miR-2 seed family have also been shown to regulate the expression of the proapoptotic genes *reaper*, *grim* and *skl* (Stark et al., 2003; Brennecke et al., 2005; Leaman et al., 2005; Thermann and Hentze, 2007) and to limit apoptosis in the developing nervous system (Ge et al., 2012).

In view of the importance of the Hippo pathway in regulating proliferation-induced apoptosis, we have examined other modes of action for Yki. Here we provide evidence for additional parallel pathways involving Yki, p53 and the miR-2 family of microRNAs in controlling the expression of *reaper* another key proapoptotic gene. Yki acts via regulation of p53 on *reaper* transcription. In some tissues, Yki acts independently via members of the miR-2 family to regulate expression of *reaper* post-transcriptionally. Our findings place Yki at the center of a network of regulatory relationships balancing cell proliferation, p53-dependent checkpoints, proapoptotic genes and miRNAs in control of tissue growth.

## Results

### Hippo pathway controls apoptosis by limiting reaper expression

**Fig. 1.**
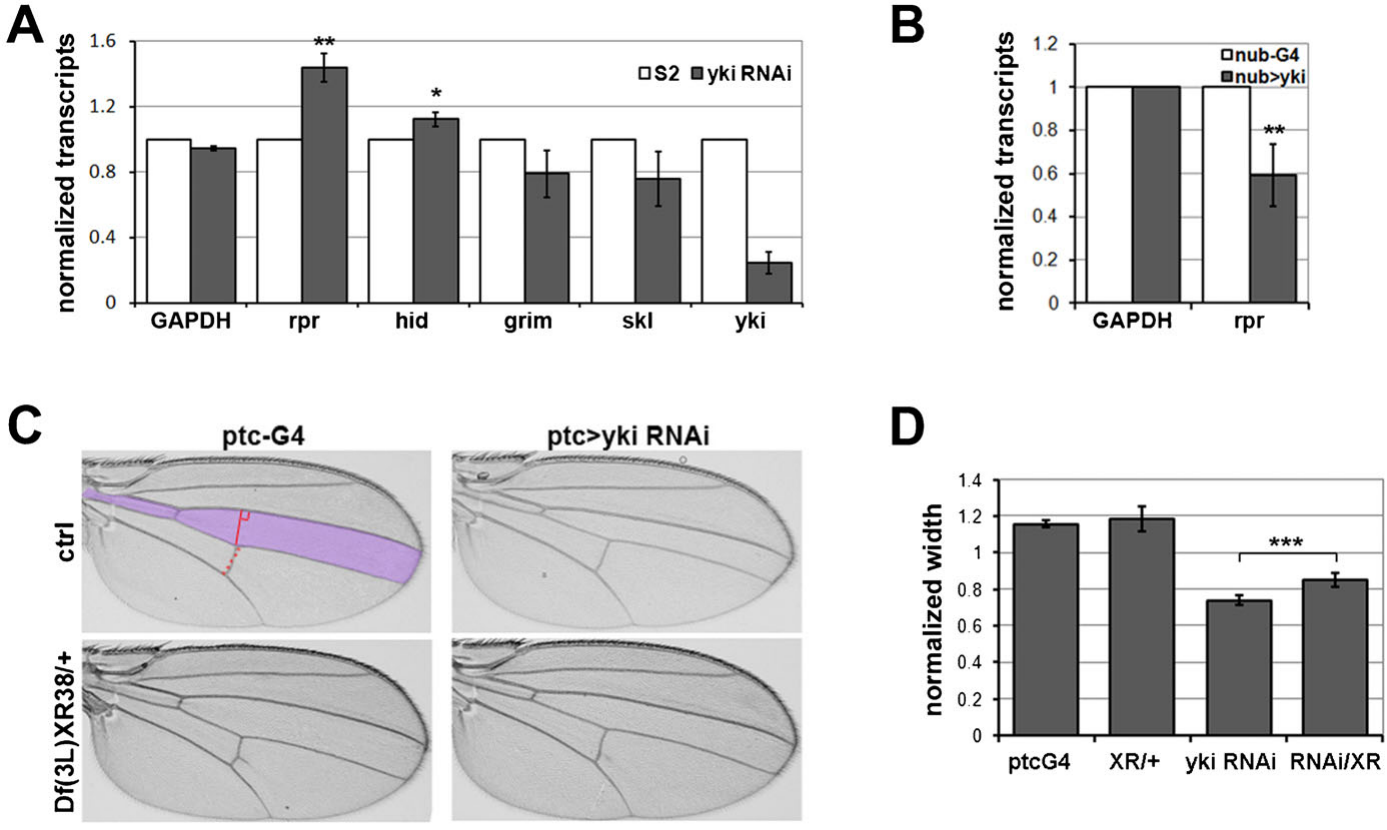
Yorkie regulates *reaper* activity in tissue growth control. (**A**) Histogram showing the levels of *reaper* (*rpr*), *hid*, *grim*, *skl*, *GAPDH* and *yki* mRNAs measured by quantitative RT-PCR. S2 cells were treated with dsRNA to deplete *yki* (grey bars) or GFP as control (white bars). The *yki* RNA measurement shows that the RNAi treatment was effective. *GAPDH* serves as a control. Total RNA was extracted and normalized for cDNA synthesis. RNA levels were normalized to *kinesin* mRNA. Error bars represent standard deviation from 3 independent experiments. (*) Student's t-test for *hid* vs *GAPDH*: *P*<0.05; (**) Student's t-test for *rpr* vs *GAPDH*: *P*<0.01. (**B**) Histogram showing the levels of *rpr* and *GAPDH* mRNAs measured by quantitative RT-PCR. RNA was extracted from wing imaginal discs expressing *nub-Gal4* alone (white bars) or *nub-Gal4* with a *UAS-Yki t*ransgene. Error bars represent standard deviation from 3 independent experiments. (**) Student's t-test for *rpr* vs *GAPDH*: *P*<0.01. (**C**) Photomicrographs of adult wings of the indicated genotype. The domain of *ptc-Gal4* expression is shaded in the upper left panel. Left panels: *ptc-Gal4* control flies. Right panels *ptc-Gal4* driving expression of a *UAS-yki^RNAi^ t*ransgene to reduce *yki* mRNA levels in the *ptc-Gal4* expression domain. Upper panels: control flies with 2 copies of the proapoptotic genes. Lower panels: flies carrying one copy of *Df(3L)XR38*. (**D**) Quantification of the effects of the treatments in panel C on the size of the *ptc-Gal4* expression domain. Data are represented as the ratio of the width of the region between veins 3–4 (solid red line) to the region between veins 4–5 (dashed red line, measured along the posterior crossvein). In normal flies this ratio is ∼1.2:1. Note that there was no effect of *ptc-Gal4* driven Yki RNAi on the size of the region between veins 4–5 (not shown), so the ratio reflects reduction of the L3–4 region. *** indicates statistically significant increase in the width of the *ptc-Gal4* expression domain when one copy of the *rpr* and *skl* genes were removed (*P*<0.001).

The transcription coactivator Yorkie mediates Hippo pathway activity to control gene expression in *Drosophila*. We used RNAi to deplete *yorkie* (*yki*) mRNA from S2 cells, to assess the contribution of the Hippo pathway to expression of genes involved in regulation of apoptosis. Depletion of *yki* mRNA was effective, and resulted in increased expression of *reaper* mRNA and a smaller increase in *hid* mRNA (Fig. 1A, **P<0.01). To test this relationship in a growing tissue *yki* was overexpressed in the wing imaginal disc under control of *nubbin-Gal4*. *yki* overexpression decreased the level of *reaper* mRNA (Fig. 1B, *P*<0.01; control for *yki* mRNA level in supplementary material Fig. S1A). Thus Yki appears to negatively regulate expression of *reaper*.

Does regulation of *reaper* contribute to the growth regulatory activity of the Hippo pathway *in vivo*? We made use of *patched-Gal4* (*ptc-Gal4*) to direct depletion of *yki* in a defined region of the wing (shaded in Fig. 1C). Expression of a *UAS-yki^RNAi^* transgene under *ptc-Gal4* control reduced the area of the relevant region of the wing (Fig. 1C). This effect was quantified by measuring the ratio of the width of the vein 3–4 region to that of the vein 4–5 region (indicated by solid and dashed red lines, upper left panel of Fig. 1C). Depletion of *yki* reduced the relative size of the region where the *Gal4* driver was expressed (Fig. 1D). This effect was partially offset by concurrently limiting *reaper* expression using a chromosomal deletion, *Df(3L)XR38*, which removes *reaper* and *skl*, but not the adjacent *grim* and *hid* genes (Peterson et al., 2002). *Df(3L)XR38* on its own showed no effect on growth, but limited the undergrowth caused by *yki* depletion (Fig. 1C,D; *P*<0.001). These observations suggest that increased expression of *reaper* contributes to the effects of *yki* depletion *in vivo*.

### Yki acts via induction of p53 activity

**Fig. 2.**
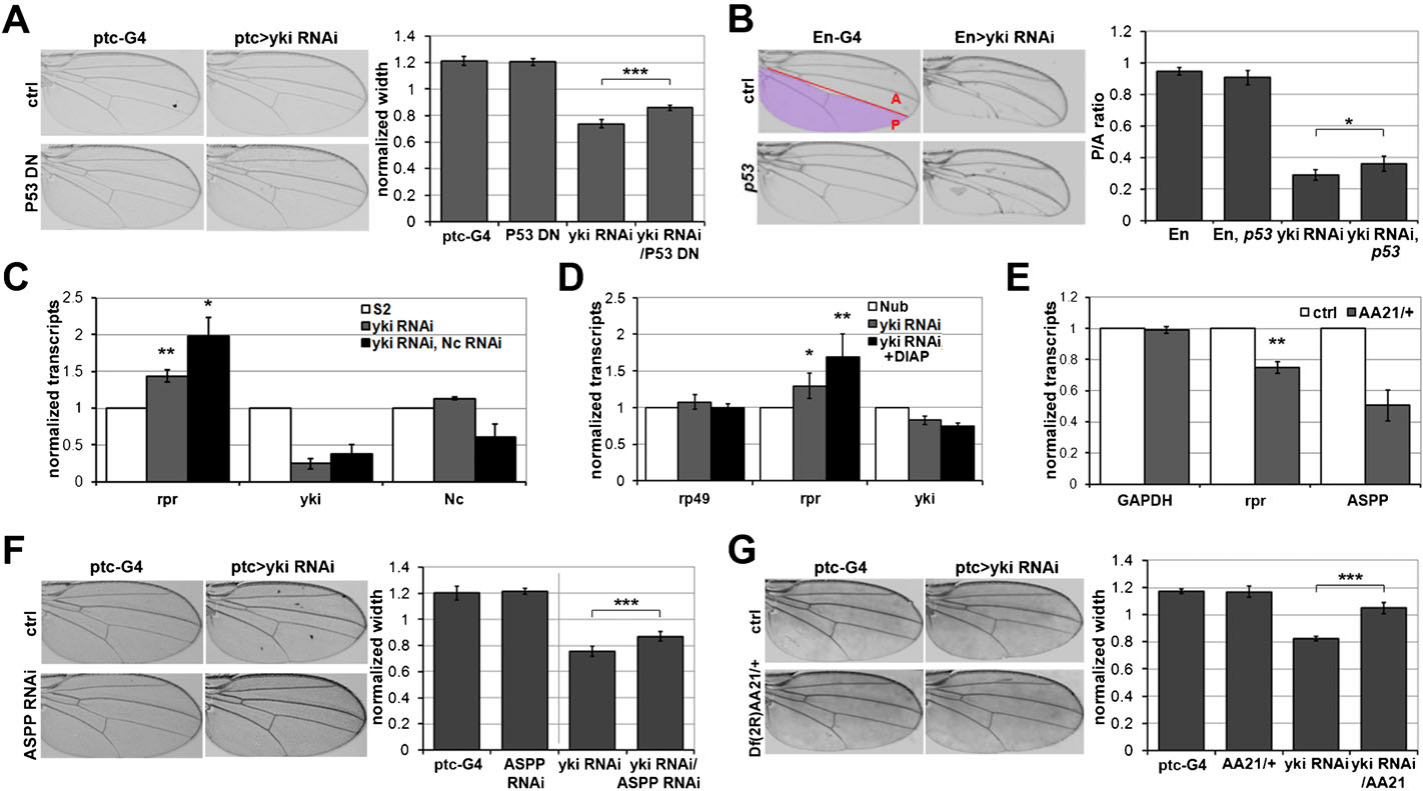
p53 mediates the effects of Yorkie. (**A**) Photomicrographs of adult wings of the indicated genotype. Left panels: *ptc-Gal4* control flies. Right panels *ptc-Gal4* driving expression of a *UAS-yki^RNAi^* transgene to reduce *yki* mRNA levels in the *ptc-Gal4* expression domain. Upper panels: control flies without expressing any other transgene. Lower panels: flies expressing a dominant negative form of p53, UAS-p53DN transgene. Histogram at right shows quantification of the effects on growth of the *ptc-Gal4* expression domain. Error bars represent standard deviation from measurement of at least 8 wings for each genotype. *** indicates statistically significant increase in the width of the *ptc-Gal4* expression domain when p53 activity was reduced (*P*<0.001). (**B**) Photomicrographs of adult wings of the indicated genotype, as in panel A, except that *en-Gal4* was used to drive transgene expression in the posterior compartment (shaded), and the ratio of anterior (A) to posterior (P) was measured. Lower panels: flies carrying two copies of a null allele of p53^5A-1-4^. Histogram shows quantification of the effects on growth of the P compartment. Error bars represent standard deviation from at least 4 wings for each genotype. * indicates statistically significant increase in the width of the *ptc-Gal4* expression domain when p53 activity was reduced (*P*<0.05). (**C**) Histogram showing the levels of *rpr*, *yki* and *Dronc (Nc)* mRNAs. S2 cells were treated with dsRNA to deplete *yki* (grey bars) or both yki and Nc (black bars) or GFP as a control (white bars). Total RNA was extracted and normalized for cDNA synthesis. RNA levels were normalized to *kinesin* mRNA. Error bars represent standard deviation from 3 independent experiments. (*) Student's t-test for *rpr* vs *GAPDH*: *P*<0.05; (**) Student's t-test for *rpr* vs *GAPDH*: *P*<0.01. (**D**) Histogram showing the levels of *rpr*, *rp49* and *yki* mRNAs measured by quantitative RT-PCR. RNA was extracted from wing imaginal discs expressing *nub-Gal4* alone (white bars) or *nub-Gal4* with *UAS-Yki RNAi* and *UAS-DIAP1 t*ransgenes. RNA levels were normalized to *kinesin* mRNA. Error bars represent standard deviation from 6 independent experiments. (*) Student's t-test for *rpr* vs *rp49*: *P*<0.05; (**) Student's t-test for *rpr* vs *rp49*: *P*<0.01. (**E**) Histogram showing the levels of *rpr*, *GAPDH* and *ASPP* mRNAs. RNA was extracted from wing imaginal discs of 3^rd^ instar control larvae (*+/+*) or *Df(2R)AA21/+* larvae (gray bars). RNA levels were normalized to *rp49* mRNA. Error bars represent standard deviation from 3 independent experiments. (**) Student's t-test for *rpr* vs *GAPDH*: *P*<0.01. (**F**) Photomicrographs of adult wings of the indicated genotype, as in panel A. Lower panels: flies expressing a *UAS-ASPP^RNAi^* transgene to reduce *ASPP* mRNA levels in the *ptc-Gal4* domain. Histogram shows quantification of the effects of the *UAS-ASPP^RNAi^* transgene alone (left) and together with *UAS-yki^RNAi^*. Left and right pairs were from separate experiments. The ratio of the L3–4 to L4–5 width is constant at ∼1.2:1 in all experiments. Error bars represent standard deviation from at least 7 wings for each genotype. *** indicates statistically significant increase in the width of the *ptc-Gal4* expression domain when ASPP activity was reduced (*P*<0.001). (**G**) Photomicrographs of adult wings of the indicated genotype, as in panel A. Lower panels: flies carrying one copy of *Df(2R)AA21*, which removes the *ASPP* gene. Error bars indicate standard deviation from at least 5 wings for each genotype. *** indicates statistically significant increase in the width of the *ptc-Gal4* expression domain when one copy of ASPP was removed (*P*<0.001).

Previous reports have shown that p53 can directly regulate *reaper* expression in *Drosophila* (Brodsky et al., 2000; Peterson et al., 2002; Zhou and Steller, 2003). This raised the possibility that Yki might act via p53 to control *reaper* during tissue growth *in vivo*. To test this we used the *ptc-Gal4 UAS-yki^RNAi^* undergrowth assay. Coexpression of a dominant negative form of p53 (p53DN) partially suppressed the tissue undergrowth caused by depletion of *yki* (Fig. 2A; *P*<0.001). Expression of p53DN on its own had no effect on growth. Similarly, reducing p53 activity by introducing a null allele of the *p53* gene also partially suppressed the effects of *engrailed-Gal4 UAS-yki^RNAi^* on tissue growth (Fig. 2B; *P*<0.05). The *p53* mutant had no effect on its own.

p53 can also be activated through the caspase Dronc (Nedd2-like caspase, Nc (Wells et al., 2006; Shlevkov and Morata, 2012)). This raised the possibility that depletion of Yki by RNAi could lead to reduced DIAP1 expression and thereby trigger Dronc-mediated activation of p53. To address this possibility, we depleted both Yki and Dronc from S2 cells and found that the increase in *reaper* mRNA levels was not reduced compared to cells depleted of Yki only, as might have been expected if the effects of Yki depletion were mediated through this feedback loop (Fig. 2C). Furthermore, *reaper* mRNA levels were higher in wing discs coexpressing *UAS-DIAP1* and *UAS-Yki^RNAi^* compared to *UAS-Yki^RNAi^* alone (Fig. 2D, *P*<0.05; DIAP1 overexpression quantified in supplementary material Fig. S1B). The increase in *reaper* levels may reflect improved survival of Yki-depleted cells when expressing DIAP1. Caspase activation due to low DIAP1 levels also seems unlikely to explain the effects of Yki depletion on *reaper* mRNA levels.

In mammalian cells expressing the oncogenic form of H-Ras, Lats, a component of the Hippo pathway, has been shown to phosphorylate ASPP1 and form a complex with ASPP1 and p53 to direct expression of pro-apoptotic genes (Aylon et al., 2010; Vigneron et al., 2010). To ask whether *Drosophila* ASPP (CG18375) might also be involved in the context of Yki regulation of p53 activity in normal tissue growth, we assessed the effects of removing one copy of the *ASPP* gene on *reaper* mRNA levels in wing discs. Quantitative RT-PCR showed that *reaper* mRNA was reduced by ∼25%, when ASPP mRNA was reduced to ∼50% in these discs (Fig. 2E; **P<0.01). Next, we assessed the effects of depleting ASPP by RNAi and the effects of removing one copy of the *ASPP* gene in the *ptc-Gal4 UAS-yki^RNAi^* undergrowth assay. In both scenarios reduced ASPP activity partially restored growth of the *yki*-depleted tissue (Fig. 2F,G; ***P<0.001).

Taken together, these observations suggest that the Hippo pathway acts through Yki and p53 to control *reaper* expression. The involvement of ASPP, suggests that this regulation is likely to be mediated through Yki binding to Lats/Wts and competing for ASPP1 phosphorylation, as described in mammalian cell culture models (Aylon et al., 2010; Vigneron et al., 2010). Here we present evidence that limiting *reaper* levels by manipulating p53-ASPP1 activity contributes to suppressing the tissue growth effects of the Hippo pathway. This observation is consistent with a model in which the Hippo pathway regulates p53 activity to control proliferation-induced apoptosis.

### Yki regulates miRNA expression to control *reaper* level

**Fig. 3.**
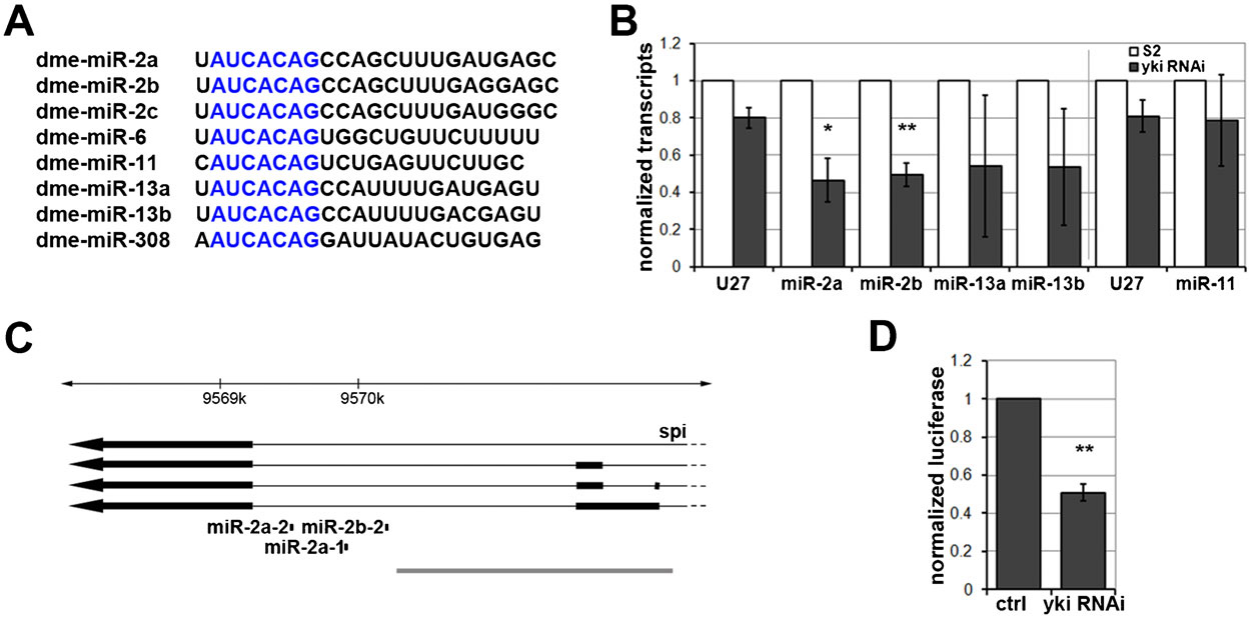
Yorkie regulates miR-2a cluster expression in S2 cells. (**A**) Sequence alignment of *Drosophila* miR-2 family miRNAs. The seed region is shown in blue to recognize *reaper* mRNA. (**B**) Histogram showing the levels of *miR-2*, *miR-13*, *miR-11* and *U27* measured by quantitative RT-PCR. S2 cells were treated with dsRNA to deplete *yki* (grey bars) or left untreated (white bars). Small RNA *U27* serves as a control. Total RNA was extracted and normalized for cDNA synthesis. RNA levels were normalized to small RNA *U14*. Gray line indicates separate experiments. The ratio of *U27* between untreated and yki RNAi sample is constant at 1:∼0.8 in all experiments. Error bars represent standard deviation from at least 3 independent experiments. Student's t-test for *miR-2* vs *U27*: *P<0.05, **P<0.01. (**C**) Schematic representation of the miR-2a cluster locus. Arrow lines represent transcripts of *spi* gene: thick parts indicate exons; thin parts indicate introns. microRNAs of miR-2 family were represented as black dots. The 1.9 kB cis-regulatory fragment directing luciferase reporter is shown as thick gray line below. (**D**) Luciferase assays showing activation of reporter directed by a 1.9 Kb DNA fragment of *miR-2a* cluster cis-regulatory element (C). S2 cells were treated with dsRNA to deplete *yki* (right bar) or GFP as a control (left bar). Error bars represent standard deviation from 3 independent experiments. (**) Student's t-test <0.01.

Previous reports have shown that microRNAs of the miR-2 seed family (Fig. 3A) can regulate *reaper, grim* and *skl* (Stark et al., 2003; Leaman et al., 2005; Brennecke et al., 2005; Thermann and Hentze, 2007). This prompted us to ask whether there might be a miRNA-based mechanism by which the Hippo pathway controls *reaper* expression. As a first step we asked which of the miR-2 family miRNAs is subject to regulation by the Hippo pathway in S2 cells. Depletion of *yki* in S2 cells by RNAi led to a significant reduction in the levels of expression of *miR-2a* and *b* (*P*<0.05, Fig. 3B; *miR-13a/b* were on average lower, but the effect was variable and so not statistically significant). *miR-11* was not significantly changed. *miR-6* is expressed at very low levels in S2 cells.

As a first step to address how Yki might regulate *miR-2* expression, we sought to identify cis-regulatory control elements that direct expression of *miR-2* loci in S2 cells. *miR-2a-1*, *miR-2a-2* and *miR-2b-2* are expressed as a cluster of 3 miRNAs located in an intron of the *spitz* gene (Fig. 3C). A 1.9 Kb DNA fragment covering the intronic sequences upstream of the miRNA cluster and spanning the next upstream exon proved sufficient to direct expression of a luciferase reporter gene in S2 cells (Fig. 3C; supplementary material Fig. S2A). We then used this luciferase reporter to assess the effects of depleting *yki* by RNAi. Expression of the *miR-2a* cluster reporter decreased significantly in *yki*-depleted cells (Fig. 3D), suggesting that Yorkie regulates transcription of the *miR-2a* cluster.

**Fig. 4.**
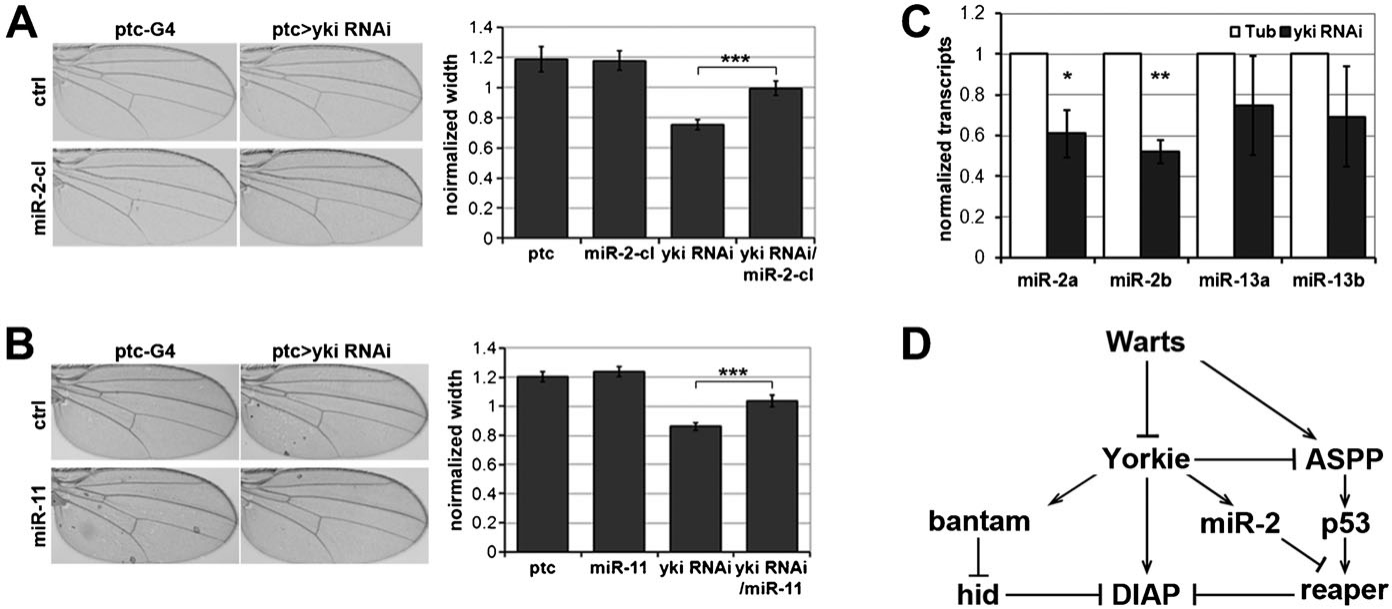
Expression of miR-2 family mediates tissue growth of Hippo pathway. (**A**) Photomicrographs of adult wings of the indicated genotype. Left panels: *ptc-Gal4* control flies. Right panels *ptc-Gal4* driving expression of a *UAS-yki^RNAi^* transgene to reduce *yki* mRNA levels in the *ptc-Gal4* expression domain. Upper panels: control flies without expressing any other transgene. Lower panels: flies expressing *UAS-miR-2a/2b* cluster transgene to increase miR-2a and 2b levels. Histogram at right shows quantification of the effects on growth of the *ptc-Gal4* expression domain. Error bars represent standard deviation from measurement of at least 8 wings for each genotype. *** indicates statistically significant increase in the width of the *ptc-Gal4* expression domain when miR-2 cluster level was increased (*P*<0.001). (**B**) Photomicrographs of adult wings of the indicated genotype, as in panel A. Lower panels: flies expressing a *UAS-miR-11* transgene to increase miR-11 level in the *ptc-Gal4* domain. Histogram shows quantification of the effects of the *UAS-miR-11* transgene alone and together with *UAS-yki^RNAi^*. Error bars represent standard deviation from at least 6 wings for each genotype. *** indicates statistically significant increase in the width of the *ptc-Gal4* expression domain when *miR-11* level was increased (*P*<0.001). As a member of the *miR-2* seed family, *miR-11* is expected to regulate the same targets as *miR-2*. Regulation of *reaper* by *miR-11* has been confirmed in the embryo by Leaman et al. and Ge et al. (Leaman et al., 2005; Ge et al., 2012). (**C**) Histogram showing the levels of *miR-2* and *miR-13* measured by quantitative RT-PCR. Wandering 3^rd^ instar larvae expressed UAS-*yki^RNAi^* under ubiquitous *tubulin-Gal4* control. Control (white bars) expressed *tubulin-Gal4* without the UAS-RNAi transgene. Total RNA from whole larvae was extracted and normalized for cDNA synthesis. RNA levels were normalized to small RNA *U14*. Error bars represent standard deviation from 3 independent experiments. Student's t-test for *miR-2* vs *U14*: *P<0.05, **P<0.01. (**D**) Yki acts at multiple levels to control apoptosis. Arrows indicate activation, whereas bar-ended lines indicate inhibitory interactions.

To further assess this regulation *in vivo*, we first asked whether overexpressing members of the miR-2 family could rescue the *ptc-Gal4 UAS-yki^RNAi^* undergrowth assay. Coexpression of a *miR-2a/2b* cluster transgene or a *miR-11* transgene suppressed the undergrowth of yki-depleted tissue caused by elevated *reaper* mRNA (Fig. 4A,B; *P*<0.001). Expression of *miR-2a/2b* or *miR-11* on their own had no effect on growth. Next we introduced a *miR-2a* sensor into the *ptc-Gal4 UAS-yki^RNAi^* assay to report *miR-2a* activity *in vivo*. The sensor transgene expresses GFP under control of the ubiquitously-expressed tubulin promoter and carries two *miR-2a* sites in its 3′ UTR (as described (Brennecke et al., 2005)). However, depletion of *yki* had no effect on the expression of the *miR-2a* reporter in wing imaginal discs (supplementary material Fig. S3). Although ectopically expressing members of *miR-2* family could suppress undergrowth of yki RNAi tissue, the negative result with the *miR-2a* reporter suggests that the effects of Yki on *reaper* are not mediated by regulation of *miR-2a* expression in the wing discs. To ask whether this regulation occurred in other tissues *in vivo*, in addition to S2 cells, we expressed UAS-*yki^RNAi^* ubiquitously under tubulin-Gal4 control and found a significant reduction of *miR-2a* and *b* in the whole 3^rd^ instar larvae (Fig. 4C; *P*<0.05). These findings suggest that the Hippo pathway contributes to control of apoptosis through regulation of *miR-2* expression in some but not all tissues.

## Discussion

Studies conducted in mammals and *Drosophila* have suggested that the downstream effectors of the Hippo pathway, YAP/TAZ and Yki direct expression of multiple targets linking cell division and cell death. Identified targets include the cell cycle regulator cycE and the cellular growth effector Myc (Huang et al., 2005; Neto-Silva et al., 2010). When the level of Hippo pathway activity is sufficient, more cycE binds to CDK2 to promote the transition from G1 to S phase promoting cell division (Hinds et al., 1992). Meanwhile, elevated Myc activates numerous target genes for ribosome assembly and cellular growth (Grewal et al., 2005). Myc activation is sufficient to induce apoptosis (Pelengaris et al., 2002), and YAP/TAZ/Yki act in parallel to limit apoptosis to ensure balance in the coordinated drive for cells to grow and divide.

Yki acts at multiple levels to control apoptosis. Yki directs expression of the *Drosophila* Inhibitor of Apoptosis Protein, DIAP1 (Huang et al., 2005). We have provided evidence that Yki acts via regulation of p53 activity to regulate *reaper* transcription. Yki acts in parallel in some tissues via regulation of miR-2 family miRNAs to regulate *reaper* activity. *miR-2* has been shown to regulate translation of *repaer* mRNA (Thermann and Hentze, 2007). In addition, Yki mediated regulation of *bantam* miRNA expression (Nolo et al., 2006; Thompson and Cohen, 2006) controls *hid* transcript levels (Brennecke et al., 2003). Thus, Yki is at the center of a network of regulatory relationships involving p53-dependent checkpoints, proapoptotic genes and expression of multiple miRNAs in control of proliferation induced apoptosis (illustrated in Fig. 4D).

Why use a variety of parallel effector mechanisms? Our findings suggest that there may be tissue-specific differences in pathway use. As well, use of multiple pathways allows for the possibility that their activity may be regulated in a manner that depends on physiological context. This may be advantageous in adapting control of growth and apoptosis to the needs of different tissue types during development and in the adult for homeostasis and tissue repair. Diverse modes of regulation may also reflect the importance of having adequate checkpoints to limit proliferation. Bypassing apoptosis and negative growth regulatory signals are important steps along the path to cancer (Hanahan and Weinberg, 2011).

## Materials and Methods

### Fly strains

*Df(3L)XR38*, which removes *rpr and skl*, but not *hid* and *grim*, is described by Peterson et al. (Peterson et al., 2002) and was provided by Kristin White. UAS-p53DN is described by Brodsky et al. (Brodsky et al., 2000). *GUS-p53DN* (p53.Ct), p53^5A-1-4^, *Df(2R)AA21* flies were obtained from the Bloomington Stock Center. *UAS-RNAi-yki* (transformant ID: 40497 and 104523) and *UAS-RNAi-ASPP* lines were from the Vienna Drosophila RNAi center. *UAS-miR-2a/2b* and *miR-2a* GFP sensor flies were described by Stark et al. (Stark et al., 2003). *UAS-miR-11* transgene was described by Szuplewski et al. (Szuplewski et al., 2012).

### Cell culture and treatments

S2 cells were grown at 25°C in SFM (Gibco) supplemented with L-glutamine. dsRNA was prepared using MegascriptT7 (Ambion) with the following templates: *yki*, nucleotides 331–875 of yki 215AA isoform coding sequence; Dronc, nt649–1122 of the ORF; GFP, nt 17–633 of EGFP2. S2 cells were treated with 37 nM dsRNA. The primers used to clone the 1.9 Kb DNA fragment before miR-2a cluster into pGL3-Basic by SLIC at XhoI site were: forward, 5′-GCGTGCTAGCCCGGGCTCGAGAAACTTTTGTTTGGTTTTGGAATATACATATATGTATGTGTG-3′; reverse, 5′-AAGCTTACTTAGATCGCAGATCTGTTTCGATTCGATGAGAGCCGAGGTG-3′. The primers used to clone the 1.5 Kb DNA fragment before miR-2b-1 using the same method were: forward, 5′-GCGTGCTAGCCCGGGCTCGAGTTTAAATGTGCTTTTTTAAATAGCGAGCCACTG-3′; reverse, 5′-AAGCTTACTTAGATCGCAGATTGAATATTGTTGACAACATGTCACTGCCAC-3′.

### Quantitative RT-PCR

Total RNA was extracted from S2 cells or wing imaginal discs and treated with DNase-1 to eliminate genomic DNA contamination. Reverse transcription to synthesize the first strand used oligo-dT primers and Superscript RT-III (Invitrogen). PCR was performed and analyzed on Applied Biosystems 7500 fast real-time PCR system. The following primers were used: yki-f, 5′-GAGCAGGCAGTTACCGAGTC-3′; yki-r, 5′-TCCATGAAGTCGTTCGATCA-3′; rpr-f, 5′-TTGCGGGAGTCACAGTGGA-3′; rpr-r, 5′-TGCGATGGCTTGCGATATTT-3′; hid-f, 5′-CCTCTACGAGTGGGTCAGGA-3′; hid-r, 5′-CGTGCGGAAAGAACACATC-3′; grim-f, 5′-TGGGAAAGGCAGGCTCAATCAAAG-3′; grim-r, 5′-ACTCGTTCCTCCTCATGTGTCC-3′; skl-f, 5′-ACCAACTTAAGCACCAACTAAGGC-3′; skl-r, 5′-TGCGCTAGTTCTCACCAACG-3′; DIAP1-f, 5′-TTGTGCAAGATCTGCTACGG-3′; DIAP1-r, 5′-CACAGCGGACACTTTGTCAC-3′; Dronc-f, 5′-GAAGTCGGCCGATATTGTGGAC-3′; Dronc-r, 5′-GCTCATTCCGGAGCTTGCTAAC-3′; ASPP-f, 5′-GACCGACGATGTCCTGTGAATATC-3′; ASPP-r, 5′-GCGACAACTGATTGCGGTACATC-3′. Kinesin, rp49 and GAPDH, which were mentioned by Zhang et al., were used as house-keeping genes (Zhang et al., 2011). Data were normalized at least to the two having similar behaviors.

For microRNA quantification, reverse transcription and PCR were performed using TaqMan® MicroRNA Assays from Applied Biosystems.

### Immunostaining and microscopy

Wandering 3rd instar larvae were collected and dissected. Tissues were fixed in PBS with 4% paraformaldehyde at room temperature for 20 min, then rinsed and washed in PBST (PBS+0.05% Triton X-100) before blocked in PBST+5% BSA. Anti-GFP and Anti-Gal4 were incubated at 4°C overnight. Secondary antibodies were incubated at room temperature for 2 hrs with DAPI. Wing imaginal discs were mounted and imaged using a Zeiss LSM700 confocal microscope.

## Supplementary Material

Supplementary Material

## References

[b1] Aylon Y. Ofir-Rosenfeld Y. Yabuta N. Lapi E. Nojima H. Lu X. Oren M. (2010). The Lats2 tumor suppressor augments p53-mediated apoptosis by promoting the nuclear proapoptotic function of ASPP1. Genes Dev. 24, 2420–2429 10.1101/gad.1954410 21041410 PMC2964752

[b2] Brennecke J. Hipfner D. R. Stark A. Russell R. B. Cohen S. M. (2003). *bantam* encodes a developmentally regulated microRNA that controls cell proliferation and regulates the proapoptotic gene hid in Drosophila. Cell 113, 25–36 10.1016/S0092-8674(03)00231-9 12679032

[b3] Brennecke J. Stark A. Russell R. B. Cohen S. M. (2005). Principles of microRNA-target recognition. PLoS Biol. 3, e85 10.1371/journal.pbio.0030085 15723116 PMC1043860

[b4] Brodsky M. H. Nordstrom W. Tsang G. Kwan E. Rubin G. M. Abrams J. M. (2000). Drosophila p53 binds a damage response element at the reaper locus. Cell 101, 103–113 10.1016/S0092-8674(00)80627-3 10778860

[b5] Cai J. Zhang N. Zheng Y. de Wilde R. F. Maitra A. Pan D. (2010). The Hippo signaling pathway restricts the oncogenic potential of an intestinal regeneration program. Genes Dev. 24, 2383–2388 10.1101/gad.1978810 21041407 PMC2964748

[b6] Dong J. Feldmann G. Huang J. Wu S. Zhang N. Comerford S. A. Gayyed M. F. Anders R. A. Maitra A. Pan D. (2007). Elucidation of a universal size-control mechanism in Drosophila and mammals. Cell 130, 1120–1133 10.1016/j.cell.2007.07.019 17889654 PMC2666353

[b7] Gartel A. L. Radhakrishnan S. K. (2005). Lost in transcription: p21 repression, mechanisms, and consequences. Cancer Res. 65, 3980–3985 10.1158/0008-5472.CAN-04-3995 15899785

[b8] Ge W. Chen Y. W. Weng R. Lim S. F. Buescher M. Zhang R. Cohen S. M. (2012). Overlapping functions of microRNAs in control of apoptosis during Drosophila embryogenesis. Cell Death Differ. 19, 839–846 10.1038/cdd.2011.161 22095284 PMC3321623

[b9] Grewal S. S. Li L. Orian A. Eisenman R. N. Edgar B. A. (2005). Myc-dependent regulation of ribosomal RNA synthesis during Drosophila development. Nat. Cell Biol. 7, 295–302 10.1038/ncb1223 15723055

[b10] Hamaratoglu F. Willecke M. Kango-Singh M. Nolo R. Hyun E. Tao C. Jafar-Nejad H. Halder G. (2006). The tumour-suppressor genes NF2/Merlin and Expanded act through Hippo signalling to regulate cell proliferation and apoptosis. Nat. Cell Biol. 8, 27–36 10.1038/ncb1339 16341207

[b11] Hanahan D. Weinberg R. A. (2011). Hallmarks of cancer: the next generation. Cell 144, 646–674 10.1016/j.cell.2011.02.013 21376230

[b12] Harvey K. F. Pfleger C. M. Hariharan I. K. (2003). The Drosophila Mst ortholog, hippo, restricts growth and cell proliferation and promotes apoptosis. Cell 114, 457–467 10.1016/S0092-8674(03)00557-9 12941274

[b13] Herranz H. Hong X. Cohen S. M. (2012). Mutual repression by bantam miRNA and Capicua links the EGFR/MAPK and Hippo pathways in growth control. Curr. Biol. 22, 651–657 10.1016/j.cub.2012.02.050 22445297

[b14] Hinds P. W. Mittnacht S. Dulic V. Arnold A. Reed S. I. Weinberg R. A. (1992). Regulation of retinoblastoma protein functions by ectopic expression of human cyclins. Cell 70, 993–1006 10.1016/0092-8674(92)90249-C 1388095

[b15] Huang J. Wu S. Barrera J. Matthews K. Pan D. (2005). The Hippo signaling pathway coordinately regulates cell proliferation and apoptosis by inactivating Yorkie, the Drosophila Homolog of YAP. Cell 122, 421–434 10.1016/j.cell.2005.06.007 16096061

[b16] Leaman D. Chen P. Y. Fak J. Yalcin A. Pearce M. Unnerstall U. Marks D. S. Sander C. Tuschl T. Gaul U. (2005). Antisense-mediated depletion reveals essential and specific functions of microRNAs in Drosophila development. Cell 121, 1097–1108 10.1016/j.cell.2005.04.016 15989958

[b17] Moberg K. H. Bell D. W. Wahrer D. C. Haber D. A. Hariharan I. K. (2001). Archipelago regulates Cyclin E levels in Drosophila and is mutated in human cancer cell lines. Nature 413, 311–316 10.1038/35095068 11565033

[b18] Moberg K. H. Mukherjee A. Veraksa A. Artavanis-Tsakonas S. Hariharan I. K. (2004). The Drosophila F box protein archipelago regulates dMyc protein levels *in vivo*. Curr. Biol. 14, 965–974 10.1016/j.cub.2004.04.040 15182669

[b19] Neto-Silva R. M. de Beco S. Johnston L. A. (2010). Evidence for a growth-stabilizing regulatory feedback mechanism between Myc and Yorkie, the Drosophila homolog of Yap. Dev. Cell 19, 507–520 10.1016/j.devcel.2010.09.009 20951343 PMC2965774

[b20] Nolo R. Morrison C. M. Tao C. Zhang X. Halder G. (2006). The bantam microRNA is a target of the hippo tumor-suppressor pathway. Curr. Biol. 16, 1895–1904 10.1016/j.cub.2006.08.057 16949821

[b21] Pan D. (2010). The hippo signaling pathway in development and cancer. Dev. Cell 19, 491–505 10.1016/j.devcel.2010.09.011 20951342 PMC3124840

[b22] Pelengaris S. Khan M. Evan G. I. (2002). Suppression of Myc-induced apoptosis in beta cells exposes multiple oncogenic properties of Myc and triggers carcinogenic progression. Cell 109, 321–334 10.1016/S0092-8674(02)00738-9 12015982

[b23] Peterson C. Carney G. E. Taylor B. J. White K. (2002). reaper is required for neuroblast apoptosis during Drosophila development. Development 129, 1467–1476. 11880355 10.1242/dev.129.6.1467

[b24] Pop C. Timmer J. Sperandio S. Salvesen G. S. (2006). The apoptosome activates caspase-9 by dimerization. Mol. Cell 22, 269–275 10.1016/j.molcel.2006.03.009 16630894

[b25] Shlevkov E. Morata G. (2012). A dp53/JNK-dependant feedback amplification loop is essential for the apoptotic response to stress in Drosophila. Cell Death Differ. 19, 451–460 10.1038/cdd.2011.113 21886179 PMC3278728

[b26] Silva E. Tsatskis Y. Gardano L. Tapon N. McNeill H. (2006). The tumor-suppressor gene fat controls tissue growth upstream of expanded in the hippo signaling pathway. Curr. Biol. 16, 2081–2089 10.1016/j.cub.2006.09.004 16996266

[b27] Stark A. Brennecke J. Russell R. B. Cohen S. M. (2003). Identification of Drosophila microRNA targets. PLoS Biol. 1, e60 10.1371/journal.pbio.0000060 14691535 PMC270017

[b28] Steller H. (2008). Regulation of apoptosis in Drosophila. Cell Death Differ. 15, 1132–1138 10.1038/cdd.2008.50 18437164

[b29] Szuplewski S. Kugler J. M. Lim S. F. Verma P. Chen Y. W. Cohen S. M. (2012). MicroRNA transgene overexpression complements deficiency-based modifier screens in Drosophila. Genetics 190, 617–626 10.1534/genetics.111.136689 22095085 PMC3276628

[b30] Tapon N. Harvey K. F. Bell D. W. Wahrer D. C. Schiripo T. A. Haber D. Hariharan I. K. (2002). salvador Promotes both cell cycle exit and apoptosis in Drosophila and is mutated in human cancer cell lines. Cell 110, 467–478 10.1016/S0092-8674(02)00824-3 12202036

[b31] Thermann R. Hentze M. W. (2007). Drosophila miR2 induces pseudo-polysomes and inhibits translation initiation. Nature 447, 875–878 10.1038/nature05878 17507927

[b32] Thompson B. J. Cohen S. M. (2006). The Hippo pathway regulates the bantam microRNA to control cell proliferation and apoptosis in Drosophila. Cell 126, 767–774 10.1016/j.cell.2006.07.013 16923395

[b33] Udan R. S. Kango-Singh M. Nolo R. Tao C. Halder G. (2003). Hippo promotes proliferation arrest and apoptosis in the Salvador/Warts pathway. Nat. Cell Biol. 5, 914–920 10.1038/ncb1050 14502294

[b34] Vigneron A. M. Ludwig R. L. Vousden K. H. (2010). Cytoplasmic ASPP1 inhibits apoptosis through the control of YAP. Genes Dev. 24, 2430–2439 10.1101/gad.1954310 21041411 PMC2964753

[b35] Wells B. S. Yoshida E. Johnston L. A. (2006). Compensatory proliferation in Drosophila imaginal discs requires Dronc-dependent p53 activity. Curr. Biol. 16, 1606–1615 10.1016/j.cub.2006.07.046 16920621 PMC1764442

[b36] Wu S. Huang J. Dong J. Pan D. J. (2003). hippo encodes a Ste-20 family protein kinase that restricts cell proliferation and promotes apoptosis in conjunction with salvador and warts. Cell 114, 445–456 10.1016/S0092-8674(03)00549-X 12941273

[b37] Yu J. Zheng Y. Dong J. Klusza S. Deng W. M. Pan D. (2010). Kibra functions as a tumor suppressor protein that regulates Hippo signaling in conjunction with Merlin and Expanded. Dev. Cell 18, 288–299 10.1016/j.devcel.2009.12.012 20159598 PMC2858562

[b38] Zhang W. Thompson B. J. Hietakangas V. Cohen S. M. (2011). MAPK/ERK signaling regulates insulin sensitivity to control glucose metabolism in Drosophila. PLoS Genet. 7, e1002429 10.1371/journal.pgen.1002429 22242005 PMC3248469

[b39] Zhao B. Wei X. Li W. Udan R. S. Yang Q. Kim J. Xie J. Ikenoue T. Yu J. Li L. (2007). Inactivation of YAP oncoprotein by the Hippo pathway is involved in cell contact inhibition and tissue growth control. Genes Dev. 21, 2747–2761 10.1101/gad.1602907 17974916 PMC2045129

[b40] Zhao B. Ye X. Yu J. Li L. Li W. Li S. Yu J. Lin J. D. Wang C. Y. Chinnaiyan A. M. (2008). TEAD mediates YAP-dependent gene induction and growth control. Genes Dev. 22, 1962–1971 10.1101/gad.1664408 18579750 PMC2492741

[b41] Zhao B. Li L. Tumaneng K. Wang C. Y. Guan K. L. (2010). A coordinated phosphorylation by Lats and CK1 regulates YAP stability through SCF(beta-TRCP). Genes Dev. 24, 72–85 10.1101/gad.1843810 20048001 PMC2802193

[b42] Zhou L. Steller H. (2003). Distinct pathways mediate UV-induced apoptosis in Drosophila embryos. Dev. Cell 4, 599–605 10.1016/S1534-5807(03)00085-6 12689597

