# Supplementary material for: The Hippo pathway acts via p53 and microRNAs to control proliferation and proapoptotic gene expression during tissue growth
Source: Biol Open. 2013 Jun 20;2(8):822–8. doi: 10.1242/bio.20134317 (PMC3744074; doi:10.1242/bio.20134317)
Supplement: Supplementary Material [file supp_2_8_822__index.html]

The Hippo pathway acts via p53 and microRNAs to control proliferation and proapoptotic gene expression during tissue growth — The Hippo pathway acts via p53 and microRNAs to control proliferation and proapoptotic gene expression during tissue growth — Supplementary Material 

# The Hippo pathway acts via p53 and microRNAs to control proliferation and proapoptotic gene expression during tissue growth

## 

**Files in this Data Supplement:**

- Supplementary Material - Wei Zhang and Stephen M. Cohen doi: 10.1242/bio.20134317
