# Supplementary material for: The Hippo pathway acts via p53 and microRNAs to control proliferation and proapoptotic gene expression during tissue growth
Source: Biol Open. 2013 Jun 20;2(8):822–8. doi: 10.1242/bio.20134317 (PMC3744074; doi:10.1242/bio.20134317)
Supplement: Supplementary Material [file supp_bio.20134317_bio.20134317-s1.pdf]

Supplementary Material

Wei Zhang and Stephen M. Cohen **doi: 10.1242/bio.20134317**

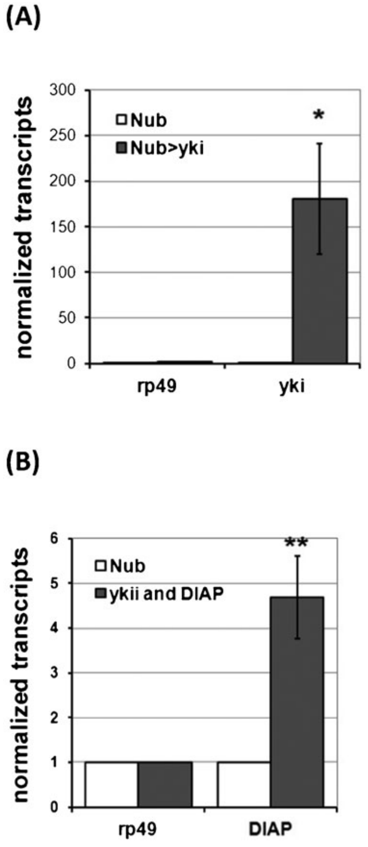

Fig. S1. Measurement of Nubbin-Gal4 driven expression of UAS transgenes. (**A**) *rp49* and *yki* mRNA measured by quantitative RT-PCR, normalized to *kinesin* mRNA (*$P<0.05$). RNA was used in Fig. 1B. (**B**) *DIAP1* mRNA measured by quantitative RT-PCR, normalized to *rp49* mRNA ($P<0.01$). RNA was used in Fig. 2D.

Biology Open

Biology Open

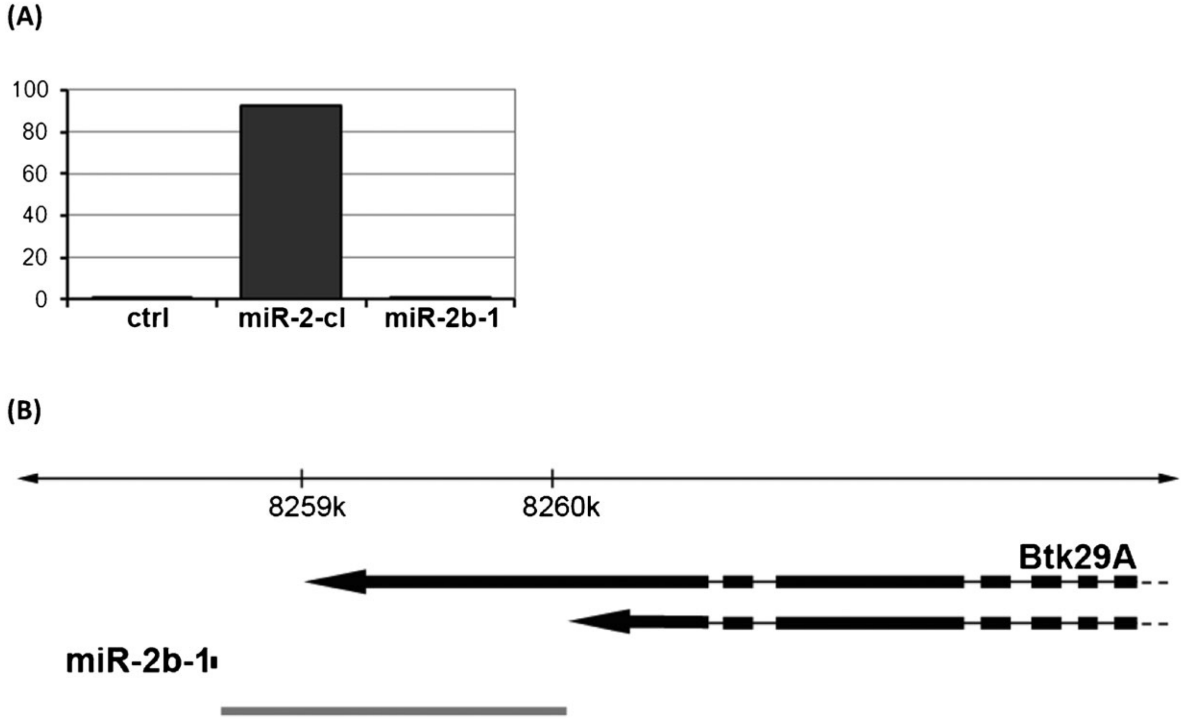

Fig. S2. Identification of a cis-regulatory element from the miR-2a cluster. (**A**) Luciferase assay showing activities of reporter plasmids after transfected into S2 cells. "miR-2-cl" denotes reporter directed by the 1.9 kB DNA fragment in Fig. 3C; "miR-2b-1" denotes reporter directed by the 1.5 kB fragment in panel B. pGL3-Basic was used as the control reporter. Data show fold induction relative to the pGL3-Basic control. (**B**) Schematic representation of the miR-2b-1 locus. Arrow lines represent transcripts of *Btk29A* gene. A 1.5 kB DNA fragment used for identification of cis-regulatory region is shown as thick gray line below. This fragment was unresponsive to Yki expression (A).

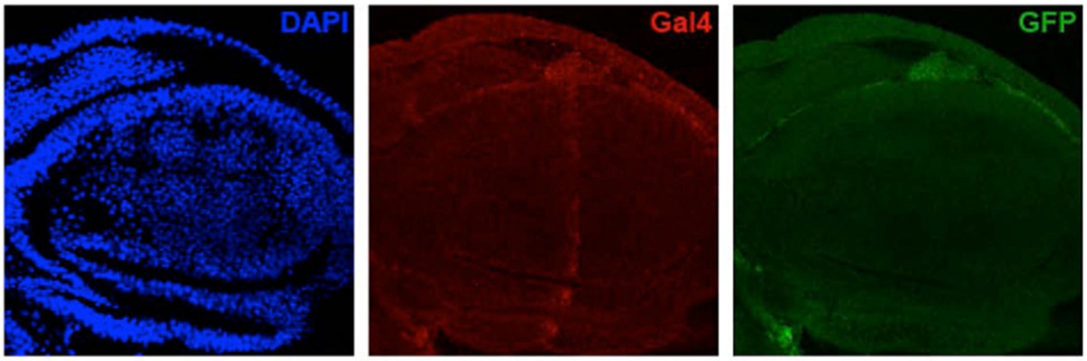

Fig. S3. Effects of Yki depletion on expression of a miR-2a sensor in the wing imaginal disc. *patched-Gal4* was used to express a $UAS\text{-}Yki^{RNAi}$ transgene in the wing imaginal disc (as described in the main text). Effects of Yki depletion were monitored using a *miR-2a* sensor transgene (as described by Brennecke et al. (Brennecke et al., 2005)). If Yki depletion reduced *miR-2a* expression, we would expect to see increased GFP expression from the sensor transgene. No change was observed.
